# Supplementary figures and images for: Isolated catatonia-like executive dysfunction in mice with forebrain-specific loss of myelin integrity
Source: eLife. 2023 Mar 9;12:e70792. doi: 10.7554/eLife.70792 (PMC9998085; doi:10.7554/eLife.70792)

Figure 1D

Prefrontal cortex

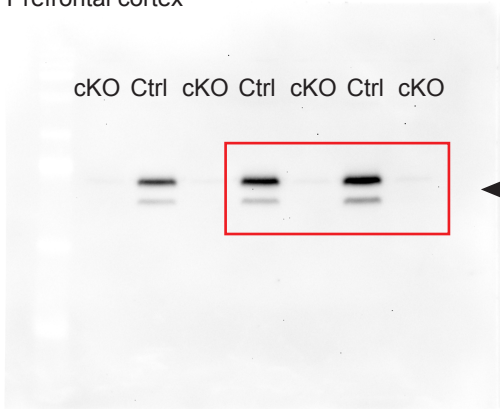

Cerebellum

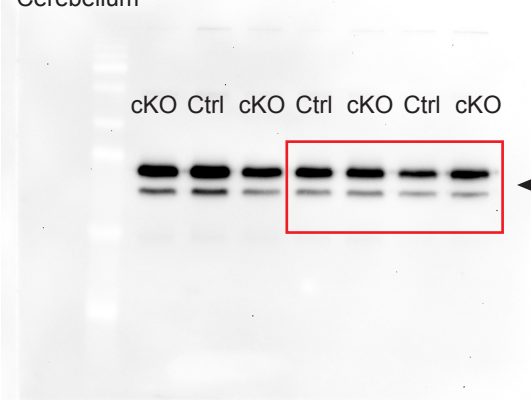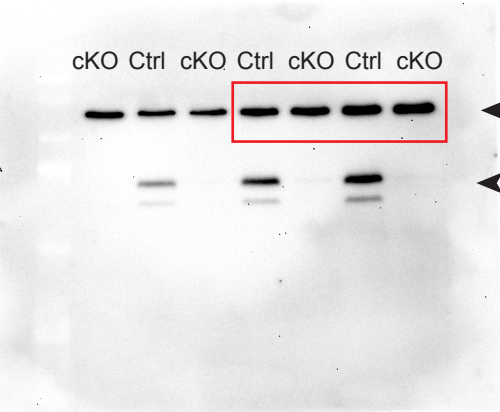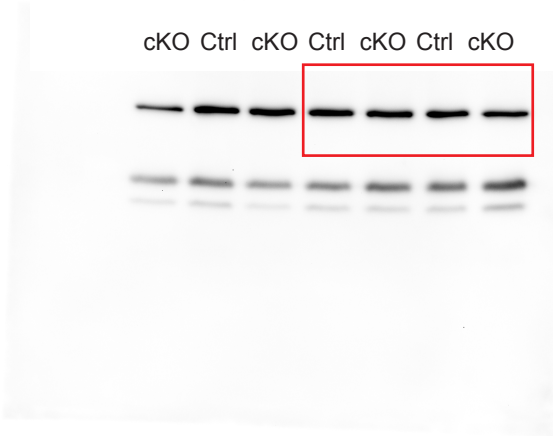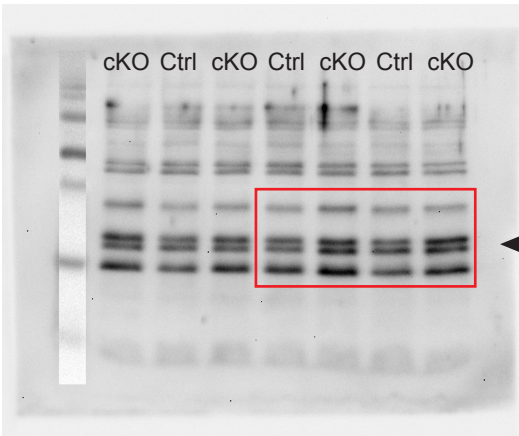

Supplement: Figure 1—source data 1. [file elife-70792-fig1-data1.zip › PLP-EMX-WB original.pdf]
