# Supplementary material for: Isolated catatonia-like executive dysfunction in mice with forebrain-specific loss of myelin integrity
Source: eLife. 2023 Mar 9;12:e70792. doi: 10.7554/eLife.70792 (PMC9998085; doi:10.7554/eLife.70792)
Supplement: Transparent reporting form [file elife-70792-transrepform1.pdf]

Westbrook Centre
Milton Road
Cambridge CB4 1YG, UK

P 01223 855340
W elifesciences.org
T @elife

eLife's transparent reporting form

We encourage authors to provide detailed information *within their submission* to facilitate the interpretation and replication of experiments. Authors can upload supporting documentation to indicate the use of appropriate reporting guidelines for health-related research (see EQUATOR Network), life science research (see the BioSharing Information Resource), or the ARRIVE guidelines for reporting work involving animal research. Where applicable, authors should refer to any relevant reporting standards documents in this form.

If you have any questions, please consult our Journal Policies and/or contact us: editorial@elifesciences.org.

Sample-size estimation

- You should state whether an appropriate sample size was computed when the study was being designed
- You should state the statistical method of sample size computation and any required assumptions
- If no explicit power analysis was used, you should describe how you decided what sample (replicate) size (number) to use

Please outline where this information can be found within the submission (e.g., sections or figure legends), or explain why this information doesn't apply to your submission:

The following information is also included in the statistics section of the manuscript:

Computation of an appropriate sample size for this study was carried out via G*Power software (Faul et al., Behavior Research Methods 2007, https://doi.org/10.3758/BF03193146) based on the following statistical requirements and assumptions: α-error 0.05, β-error 0.085, statistical effect size 1.000, based on previous IntelliCage-based experiments in our lab. We chose the IntelliCage-based assay serving a as a crucial limiting experiment, due to the high cognitive demand of the setup (see also: Dere et al., Neurobiology of learning and memory 2018, doi: 10.1016/j.nlm.2018.02.023.)

Replicates

- You should report how often each experiment was performed
- You should include a definition of biological versus technical replication
- The data obtained should be provided and sufficient information should be provided to indicate the number of independent biological and/or technical replicates
- If you encountered any outliers, you should describe how these were handled
- Criteria for exclusion/inclusion of data should be clearly stated
- High-throughput sequence data should be uploaded before submission, with a private link for reviewers provided (these are available from both GEO and ArrayExpress)

Please outline where this information can be found within the submission (e.g., sections or figure legends), or explain why this information doesn't apply to your submission:

eLife Sciences Publications, Ltd is a limited liability non-profit non-stock corporation incorporated in the State of Delaware, USA, with company number 5030732, and is registered in the UK with company number FC030576 and branch number BR015634 at the address Westbrook Centre, Milton Road, Cambridge CB4 1YG.

All information regarding experimental details is provided within the material and methods section. A detailed list of the behavioral experiments is provided in supplementary table 1. The following text is part of the statistics section:

Mice of both genders (male/female) and both genotypes (control/conditional mutant) were used, with each mouse being a biological replicate in each assay. Statistical outliers were assessed using the GraphPad Grubbs outlier tool, but no such outliers had to be removed from the data. Only mice of any gender or genotype that did not perform in an experiment were excluded from statistical analyses.

Statistical reporting

- Statistical analysis methods should be described and justified
- Raw data should be presented in figures whenever informative to do so (typically when N per group is less than 10)
- For each experiment, you should identify the statistical tests used, exact values of N, definitions of center, methods of multiple test correction, and dispersion and precision measures (e.g., mean, median, SD, SEM, confidence intervals; and, for the major substantive results, a measure of effect size (e.g., Pearson's r, Cohen's d)
- Report exact p-values wherever possible alongside the summary statistics and 95% confidence intervals. These should be reported for all key questions and not only when the p-value is less than 0.05.

Please outline where this information can be found within the submission (e.g., sections or figure legends), or explain why this information doesn't apply to your submission:

All information regarding statistical analyses and the actual scatter of individual mice, the exact N numbers and p-values are provided within the material and methods section, the respective figures and figure legends and in supplementary table 1.

(For large datasets, or papers with a very large number of statistical tests, you may upload a single table file with tests, Ns, etc., with reference to sections in the manuscript.)

Group allocation

- Indicate how samples were allocated into experimental groups (in the case of clinical studies, please specify allocation to treatment method); if randomization was used, please also state if restricted randomization was applied
- Indicate if masking was used during group allocation, data collection and/or data analysis

Please outline where this information can be found within the submission (e.g., sections or figure legends), or explain why this information doesn't apply to your submission:

Allocation of samples into respective experimental groups was based on gender (male vs female mice) and genotype (control vs conditional knockout mice). This information can be found within the materials and methods section.

Additional data files ("source data")

- We encourage you to upload relevant additional data files, such as numerical data that are represented as a graph in a figure, or as a summary table

eLife Sciences Publications, Ltd is a limited liability non-profit non-stock corporation incorporated in the State of Delaware, USA, with company number 5030732, and is registered in the UK with company number FC030576 and branch number BR015634 at the address Westbrook Centre, Milton Road, Cambridge CB4 1YG.

- Where provided, these should be in the most useful format, and they can be uploaded as “Source data” files linked to a main figure or table
- Include model definition files including the full list of parameters used
- Include code used for data analysis (e.g., R, MatLab)
- Avoid stating that data files are “available upon request”

Please indicate the figures or tables for which source data files have been provided:

Data sharing plan: All primary data, including uncropped Western blots (Fig. 1), and the raw data from histological images (Fig. 2, Suppl. Fig 2) and the behavioural experiments (Figs 3-4, Suppl. Fig 3) will be provided as digital images and Excel Tables, respectively, once the paper is accepted. The many GB data volumes from light sheet microscopy will then be available on request.

eLife Sciences Publications, Ltd is a limited liability non-profit non-stock corporation incorporated in the State of Delaware, USA, with company number 5030732, and is registered in the UK with company number FC030576 and branch number BR015634 at the address Westbrook Centre, Milton Road, Cambridge CB4 1YG.
